# Long-term follow-up of conservative treatment of Charcot feet

**DOI:** 10.1007/s00402-021-03881-5

**Published:** 2021-04-07

**Authors:** Viviane Gratwohl, Thorsten Jentzsch, Madlaina Schöni, Dominik Kaiser, Martin C. Berli, Thomas Böni, Felix W. A. Waibel

**Affiliations:** Divisions of “Prosthetics and Orthotics” and “Foot and Ankle Surgery”, Department of Orthopedics, Balgrist University Hospital, Forchstrasse 340, 8008 Zürich, Switzerland

**Keywords:** Charcot, Neuroarthropathy, Long-term follow-up, Conservative treatment, Ulceration, Amputation, Recurrence

## Abstract

**Background:**

Charcot arthropathy (CN) can ultimately lead to limb loss despite appropriate treatment. Initial conservative treatment is the accepted treatment in case of a plantigrade foot. The aim of this retrospective study was to investigate the mid- to long-term clinical course of CN initially being treated conservatively, and to identify risk factors for reactivation and contralateral development of CN as well as common complications in CN.

**Methods:**

A total of 184 Charcot feet in 159 patients (median age 60.0 (interquartile range (IQR) 15.5) years, 49 (30.1%) women) were retrospectively analyzed by patient chart review. Rates of limb salvage, reactivation, contralateral development and common complications were recorded. Statistical analysis was performed to identify possible risk factors for limb loss, CN reactivation, contralateral CN development, and ulcer development.

**Results:**

Major amputation-free survival could be achieved in 92.9% feet after a median follow-up of 5.2 (IQR 4.25, range 2.2–11.25) years. CN recurrence occurred in 13.6%. 32.1% had bilateral CN involvement. Ulcers were present in 72.3%. 88.1% patients were ambulating in orthopaedic footwear without any further aids. Presence of Diabetes mellitus was associated with reactivation of CN, major amputation and ulcer recurrence. Smoking was associated with ulcer development and necessity of amputations.

**Conclusions:**

With consistent conservative treatment of CN with orthopaedic footwear or orthoses, limb preservation can be achieved in 92.9% after a median follow-up of 5.2 years. Patients with diabetic CN are at an increased risk of developing complications and CN reactivation. To prevent ulcers and amputations, every effort should be made to make patients stop smoking.

**Level of Evidence:**

III, long-term retrospective cohort study

**Supplementary Information:**

The online version contains supplementary material available at 10.1007/s00402-021-03881-5.

## Introduction

Charcot arthropathy (CN) has first been described in 1868 by Jean-Martin Charcot [1]. As a chronic, often progressive disease, it affects mostly bones and joints, but also the surrounding soft tissues, and places the affected limb at an increased risk of lower extremity amputation [2, 3]. The incidence of CN is 0.12–0.3% in diabetic patients [3–5]. At the time of Charcot`s first description, tabes dorsalis was thought to be the reason for CN [1]. Today, presence of polyneuropathy is known to be the prerequisite for diagnosis of CN [6]. Diabetic neuropathy has become the main risk factor to develop CN aside of the alcoholic polyneuropathy, kidney failure, spinal cord injury or other neuropathies [7]. The Charcot foot appears clinically mostly painless, swollen, flushed and overheated [8]. On conventional X-rays, there is a bony destruction, which typically progresses and results in foot deformities [9]. Those deformities can cause a variety of complications such as ulcers, soft tissue infection or osteomyelitis, with subsequent surgical treatment including amputation [10].

Common classifications of CN exist for clinical stages and anatomical patterns.

The Eichenholtz classification describes three clinical and imaging stages merging into each other [11]. Stage 1 is the active stage with destruction and fragmentation. Stage 2 is the healing stage with coalescence, sclerosis und sintering. Stage 3 is the chronic disease with completed bone remodeling. The original classification was later modified by Shibata et al. with an overheated, swollen, flushed foot with normal radiographic findings, but bone edema and stress fractures in MRI [12].

The Sanders and Frykberg classification defines five anatomical types of [13]. Type I involves the forefoot joints, Type II involves the Lisfranc’s joints, the tarsometatarsal joint, including the metatarsal bases, cuneiforms, and cuboid, Type III includes Chopart’s joints or the naviculocuneiform joints, Type IV contains the ankle with or without subtalar joint involvement and Type V involves the calcaneus [13].

The diagnosis of CN is made based on clinical signs supported by radiological imaging [8, 14]. Presence of polyneuropathy or small fibre neuropathy is an indispensable condition to diagnose CN [6, 15]. In case of ambiguity or to exclude differential diagnoses such as infections, ulcerations, osteomyelitis, joint instability or fractures, MRI is recommended because of the highest diagnostic accuracy [8]. Laboratory investigations can help differentiating between CN and infection [14]. However, C-reactive protein, white blood cell count and erythrocyte sedimentation rate may also be elevated in active CN compared to non-active CN [16].

The aim of treatment is to save the affected limb and to maintain or restore mobility [2, 8]. While there is a trend for more aggressive surgical treatment to achieve a plantigrade foot, the preferred treatment for active CN is activity reduction and off-loading in a total contact cast [8, 14, 17, 18]. Off-loading is upheld until swelling and erythema have vanished and the difference in temperature is less than 2° Celsius compared to the contralateral foot [14, 18]. Protected weightbearing leads to a lower number of complications compared to unprotected weightbearing [19]. After transition to Eichenholtz stage 3, further treatment consists of individual orthopaedic shoes and prevention of complications. If conservative treatment fails or if it is impossible due to a non-plantigrade foot or instability, surgical treatment is indicated [8, 14, 20]. Most often, indications are recurrent ulcers, malalignement and soft tissue infection or even osteomyelitis. Procedures that are performed are simple ulcer debridements, open reductions, exostosectomies, arthrodesis with internal or external fixation, or finally amputations [8, 14, 20–23]. Amputations can be divided into minor and major amputations with the latter being procedures above the ankle joint [24]. In his benchmark analysis, Pinzur reported that 48.5% of CN patients can be treated conservatively [2].

But what is the clinical course of patients being treated conservatively? Complications such as ulcers, ulcer recurrence, amputation, CN reactivation, and contralateral CN involvement were investigated several times and showed a large variety of results. Ulcers occur in 37–67% [4, 25–31] and ulcer recurrence in 40–49% of cases [29, 30]. Amputation rates range from 8.9 to 25.7% and the annual amputation rate is 2.7% [23, 27, 30]. Reactivation of CN has been seen in 7.1–33% [4, 32, 33]. The contralateral foot is affected in 9.6% to 30% by CN [32, 33]. While there is a multitude of literature on short-term specific questions concerning CN, there is only little evidence with rather small cohorts on mid- to long-term clinical follow-up for patients being treated for CN initially conservatively [4, 25, 27–31, 34, 35]. None of these studies contains information on all of the above-mentioned complications of CN.

Therefore, the aim of this study was to describe the long-term outcome of patients being treated for CN initially conservatively. Primary outcome measure was the amount of major amputation-free survival. Secondary outcome measures were CN complications (ulcers, ulcer recurrence, CN reactivation, contralateral CN development) and surgical interventions. Further, we sought to determine risk factors for the occurrence of the above-mentioned CN complications as well as reactivation and contralateral CN development. Finally, we aimed to compare our results to literature on surgical treatment of CN. In the author`s opinion, it is appropriate to categorize these results as “long-term” as the study contains data of up to 20 years follow-up.

## Patients and methods

Balgrist University Hospital is a tertiary orthopaedic surgery referral center with a specialized unit for “Technical Orthopaedics” (treatment of CN, diabetic feet, chronic wounds, PAD complications, and amputations as well as prosthetics and orthotics).

Patient’s data were retrospectively collected from medical records of patients during the period from January 1st 1995 to March 31st 2018 from the hospital's own information system. All patients were treated by or under direct supervision of one senior orthopaedic surgeon (T.B.). This study was approved by the local Research Ethics Committee (BASEC-Number 2018-00166).

Inclusion criteria were first time diagnosis of CN at the time of admission (by the Eichenholtz criteria [11] and confirmation by conventional X-rays) and conservative treatment initially, age > 18 years, length of clinical follow-up of at least 2 years and informed patient consent obtained in accordance to the rules of the local Research Ethics Committee. Exclusion criteria were primarily surgically treated patients with CN due to a non-plantigrade foot, patients with insufficient documentation in the medical history, and clinical follow-up of less than 2 years. 253 patients were identified. 38 patients refused study participation and were excluded, 56 patients had inadequate patient documentation. In consequence, 159 patients with 184 affected feet were included in the study.

Primary outcome measure was the major amputation-free survival. Secondary outcome measures were CN reactivation, contralateral CN development, rates of ulcers and ulcer recurrence, rates of minor amputations and other surgical procedures related to the diagnosis of CN.

On admission, diagnosis of CN was made using the Eichenholtz criteria [11] and confirmed by conventional X-rays. Decision on conservative vs. surgical treatment was made under the supervision of T.B. Patients who needed immediate surgical correction were not analysed in this study. Patients with a non-collapsed plantigrade CN foot were conducted to conservative therapy. In 119 (64.7%) feet, a total contact cast treatment was initiated which lasted on average 191 days (range 7–640 days) due to CN activity (Eichenholtz stage one or two) or ulcer presence in need of offloading. Total contact casting was performed by one of three nurses who have been trained in casting diabetic and Charcot feet throughout the whole observation period. In the remaining 65 (35.3%) feet, orthopedic custom-made shoes or orthopedic serial-made shoes could be fitted immediately after diagnosis due to Eichenholtz stage three at initial presentation or patient`s refusal of total contact casting.

After total contact casting ended, 112 (60.9%) feet were provided with orthopedic custom-made shoes, 5 (2.7%) feet with orthopedic custom made shoes including an orthotic element for the purpose of hindfoot stabilization, 45 (24.5%) feet with orthopedic serial-made shoes, 2 (1.1%) feet with normal shoes (refusal of any kind of orthopaedic shoes), 5 (2.7%) feet with an ankle foot orthosis (AFO), 1 (0.5%) foot with a therapeutic shoe (patient`s own choice due to the ease of use), and 1 (0.5%) foot with a total contact cast (patient`s own choice instead of a recommended AFO). 13 (7.1%) feet needed a lower leg prosthesis due to major amputation. Besides the two patients (both had one CN foot) who refused any kind of orthopaedic footwear and 13 (7.1%) feet undergoing major amputation, all remaining 169 feet were fitted with a custom molded depth-insole by a certified orthopaedic shoemaker (Swiss master craftsman education). CN reactivation was defined as bony reaffection diagnosed by the clinical criteria of Eichenholtz [11] and confirmed by conventional X-rays and MRI.

Statistical analysis was performed using STATA/IC (version 13; Stata Corp., College Station, TX, USA). The Chi-squared and Wilcoxon rank sum tests were used for comparison of groups. Kaplan–Meier survival estimates were calculated for CN reactivation and for contralateral CN development. Further, Kaplan–Meier survival estimates were calculated for amputation-free survival using SPSS (IBM Corp., Version 26, Armonk, NY, USA). A two-tailed T-Test was used to compare our primary and secondary outcome measures to the results of surgical literature. Values with *p* < 0.05 were considered statistically significant.

## Results

Patient`s demographics are summarized in Table 1. 49 (30.8%) patients were female, 77 (41.8%) feet were left feet. 21 (13.2%) patients died during follow-up after a median follow-up of 5.2 (IQR (interquartile range) 4.25, range 2.2–11.25) years after initiation of treatment. Median age at the time of first diagnosis of CN was 60.0 (IQR 15.5) years.

**Table 1:** Patients demographics (*n* = 159)

Variable	Median (IQR)
Age (years)	60 (15.5)
Duration of follow-up (years)	5.2 (4.25, range 2.2–11.25)
BMI (kg/m^2^)	28.7 (2.7)

Variable	Number (percent)
Women	49 (30.8%)
Death (n)	21 (13.2%)
Diabetes	115 (72.3%)
Type 1	17 (10.7%)
Type 2	98 (61.6%)
Smoker	49 (30.8%)
pAVK (n)	36 (22.6%)
Chronic kidney failure	36 (22.6%)
Cause of Neuropathy	
Diabetic	129 (70.1%)
Vitamin B12 deficiency	4 (2.2%)
Pharmacotoxic	1 (0.5%)
Ethyltoxic	16 (8.7%)
Spinal	4 (2.2%)
Inflammatory	3 (1.6%)
Idiopathic	27 (14.7%)

### Clinical stage and sanders type

According to the modified Eichenholtz classification [11, 12], 12 (6.5%) feet were in the prodromal stage, 89 (48.4%) feet in stage 1, 18 (9.8%) feet in stage 2 and 53 (28.8%) feet in stage 3. 12 (6.5%) feet were at the stage of infection. Table 2 summarizes clinical and anatomical distribution at initial diagnosis and at reactivation.

**Table 2:** Clinical and anatomical stage initially and at reactivation

	Initial (* n* = 184)	Reactivation (* n* = 25)
Clinical stage (mod. Eichenholtz [12, 40])		
Stage 0	12 (6.5%)	0 (0%)
Stage 1	89 (48.4%)	20 (80.0%)
Stage 2	18 (9.8%)	5 (20.0%)
Stage 3	53 (28.8%)	0 (0%)
Infection	12 (6.5%)	0 (0%)
Anatomical stage (Sanders and Frykberg [38])		
Type 1	25 (13.6%)	1 (4.0%)
Type 2	87 (47.3%)	6 (24.0%)
Type 3	56 (30.4%)	6 (24.0%)
Type 4	11 (6.0%)	9 (36.0%)
Type 5	5 (2.7%)	3 (12.0%)

### Reactivation

**Fig. 1:**
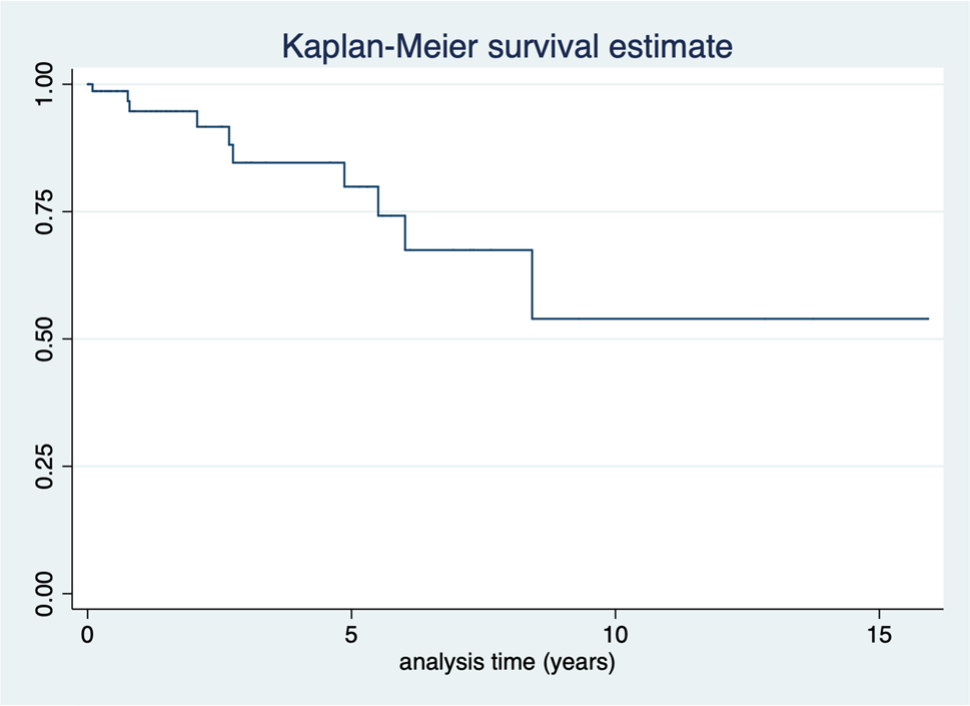
Reactivation free survival

In 25 (13.6%) feet, reactivation was detected clinically and confirmed by MRI. Median time to reactivation was 1.9 (IQR 2.2) years. Clinical and anatomical distributions are given in Table 2 while the Kaplan–Meier survival estimate for reactivation-free survival is given in Fig. 1.

### Contralateral Charcot development

**Fig. 2:**
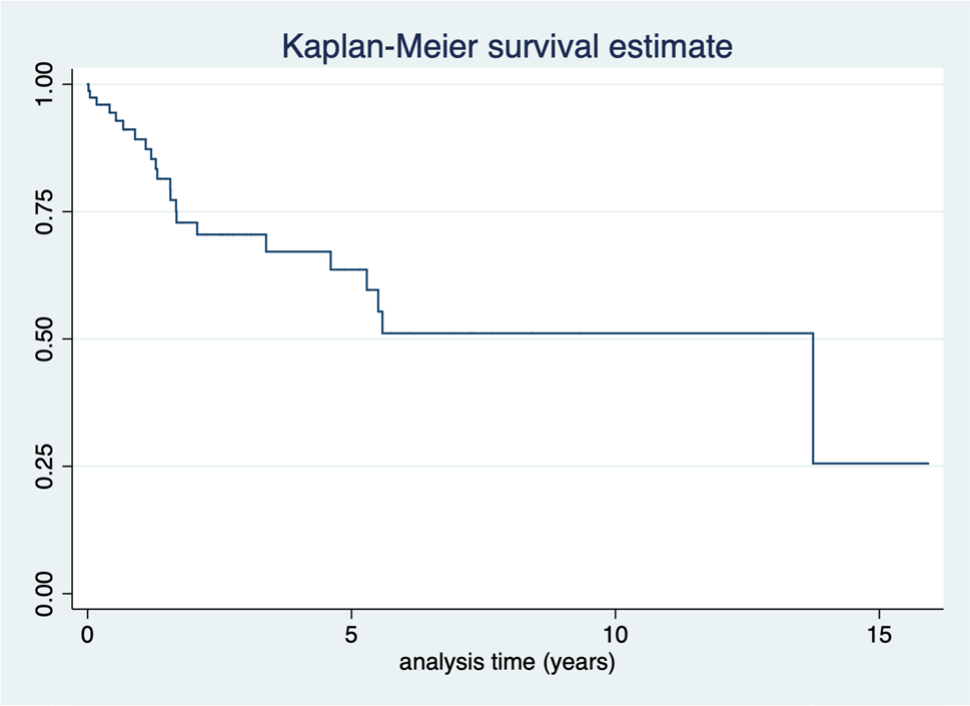
Contralateral CN free survival

133 (83.6%) patients had unilateral CN at the time of initial diagnosis, 26 (16.4%) patients were affected bilaterally. 25/133 (18.8%) patients developed CN on the contralateral side during follow-up. Median latency to contralateral CN development was 1.1 (IQR 2.8) years. In summary, 51/159 (32.1%) patients had bilateral CN at the end of follow-up. Of note, three patients presented initially with contralateral major amputation without having contralateral CN prior to amputation. Kaplan–Meier survival estimate for contralateral CN development-free survival is given in Fig. 2.

### Mobility

119 (74.8%) patients were still able to walk six blocks (483 m) without any aids at the end of the follow-up. 37 (23.3%) patients were able to walk six blocks with aids only (walking sticks, walker). Two (1.3%) patients were wheelchair-bound while one (0.6%) was bed-ridden.

### Ulcers and ulcer recurrence

At least one ulcer occurred in 133 (72.3%) feet during follow-up: in 55 (29.9%) feet at first diagnosis and in 78 (42.4%) feet during follow-up. Median time to first time ulceration was 1.4 (IQR 2.4) years when patients presented without any ulcer initially. Ulcer distribution is shown in Table 3. A total of 89 (48.4%) patients had ulcer recurrence. The median latency between initial treatment and ulcer recurrence was 1.7 (IQR 3.3) years.

**Table 3:** Localization of ulceration at ulcer initial and ulcer recurrence and percentage of ulceration by Sanders Type

	First time ulcer (*n* = 133)	Ulcer recurrence (*n* = 89)
Localization of ulceration		
Toe level^a^	84 (63.2%)	60 (67.4%)
Lisfranc level^b^	26 (19.5%)	16 (18%)
Chopart level^b^	14 (10.5%)	9 (10.1%)
Heel^b^	9 (6.8%)	4 (4.5%)

Percentage of ulceration per Sanders Type	n (%)	
Type 1 (* n* = 25)		
Toe level	17 (68%)	14 (56%)
Lisfranc level	2 (8%)	0 (0%)
Chopart level	1 (4%)	0 (0%)
Heel level	1 (4%)	0 (0%)
Type 2 (*n* = 87)		
Toe level	40 (46%)	35 (40%)
Lisfranc level	19 (21.8%)	12 (13.8%)
Chopart level	8 (9.2%)	4 (4.6%)
Heel level	0 (0%)	2 (2.3%)
Type 3 (*n* = 56)		
Toe level	22 (39.3%)	10 (17.8%)
Lisfranc level	5 (8.9%)	4 (7.1%)
Chopart level	5 (8.9%)	5 (8.9%)
Heel level	0 (0%)	1 (1.8%)
Type 4 (*n* = 11)		
Toe level	4 (36.4%)	0 (0%)
Lisfranc level	0 (0%)	0 (0%)
Chopart level	0 (0%)	0 (0%)
Heel level	1 (9.1%)	0 (0%)
Type 5 (*n* = 5)		
Toe level	1 (20%)	1 (20%)
Lisfranc level	0 (0%)	0 (0%)
Chopart level	0 (0%)	0 (0%)
Heel level	2 (40%)	1 (20%)

^a^Plantar toe tip or dorsal PIP or DIP ulcers due to claw toe deformity

^b^Plantar ulcers

### Surgical interventions

78 (42.4%) feet needed one or multiple CN-related surgical procedures during follow-up. The median latency between first consultation and surgery was 1.5 (IQR 4.1) years. One patient, who presented with an ulcer but without signs of infection initially, developed unexpected fulminant infection just 4 days after initial diagnosis and therefore, transtibial amputation had to be performed.

**Fig. 3:**
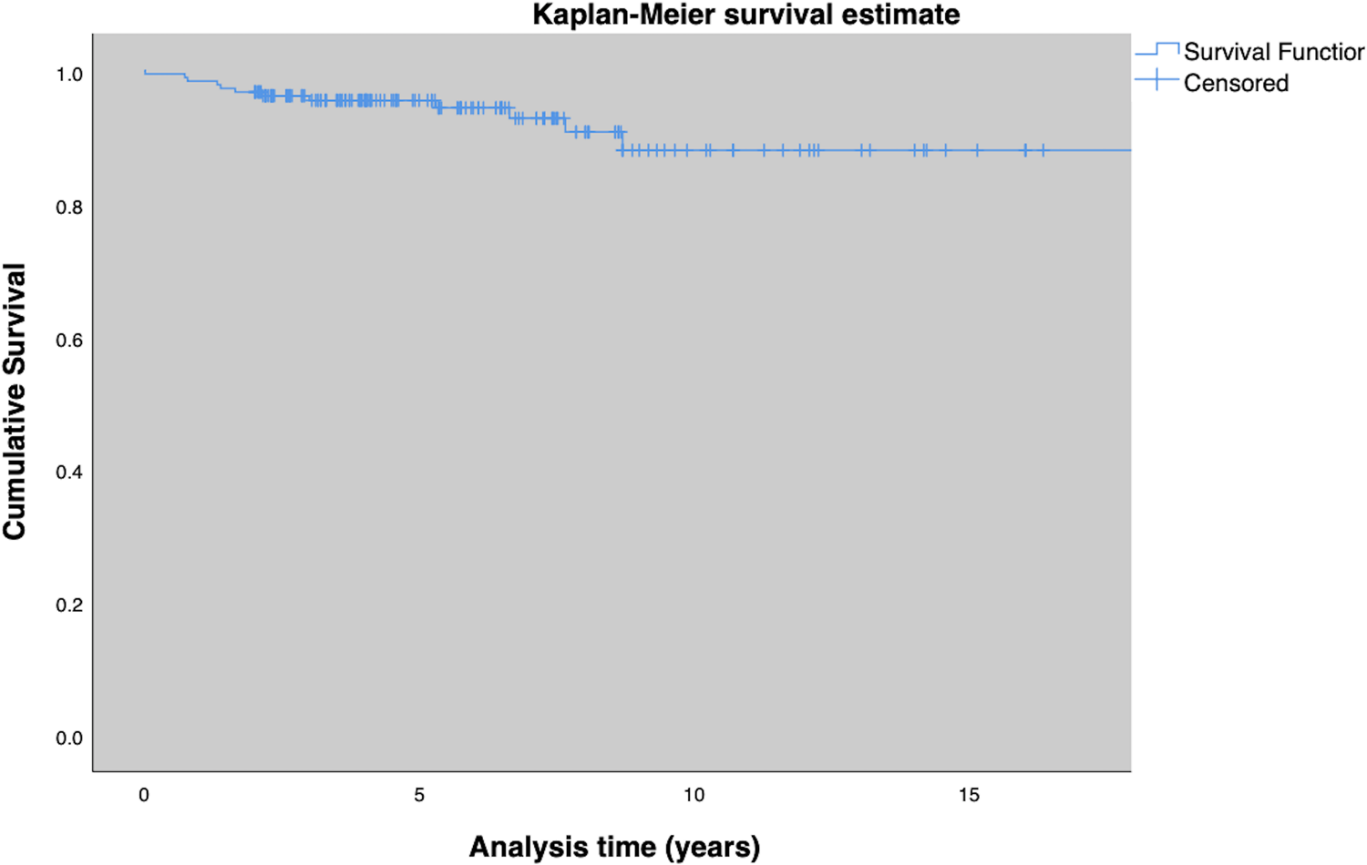
Amputation free survival for the entire cohort

**Fig. 4:**
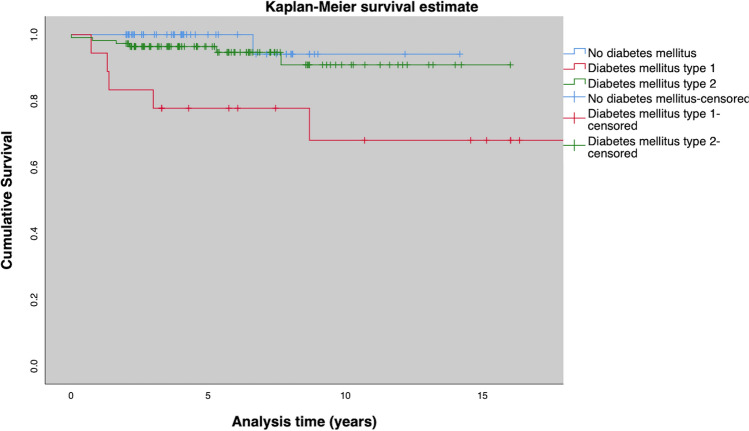
Amputation free survival for patients without Diabetes, type 1 and type 2 diabetes

**Fig. 5:**
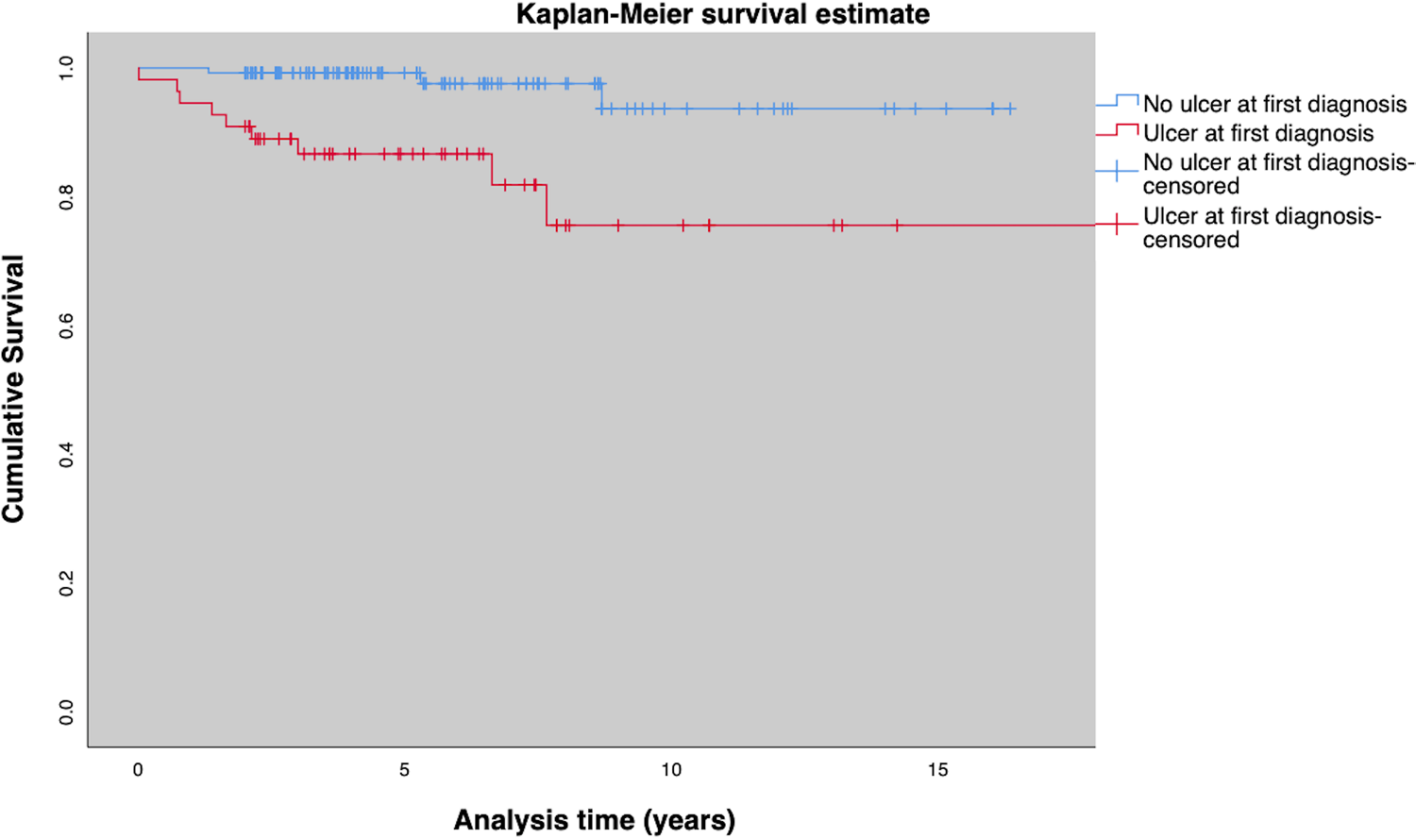
Amputation free survival for patients with and without an ulcer at initial presentation

**Fig. 6:**
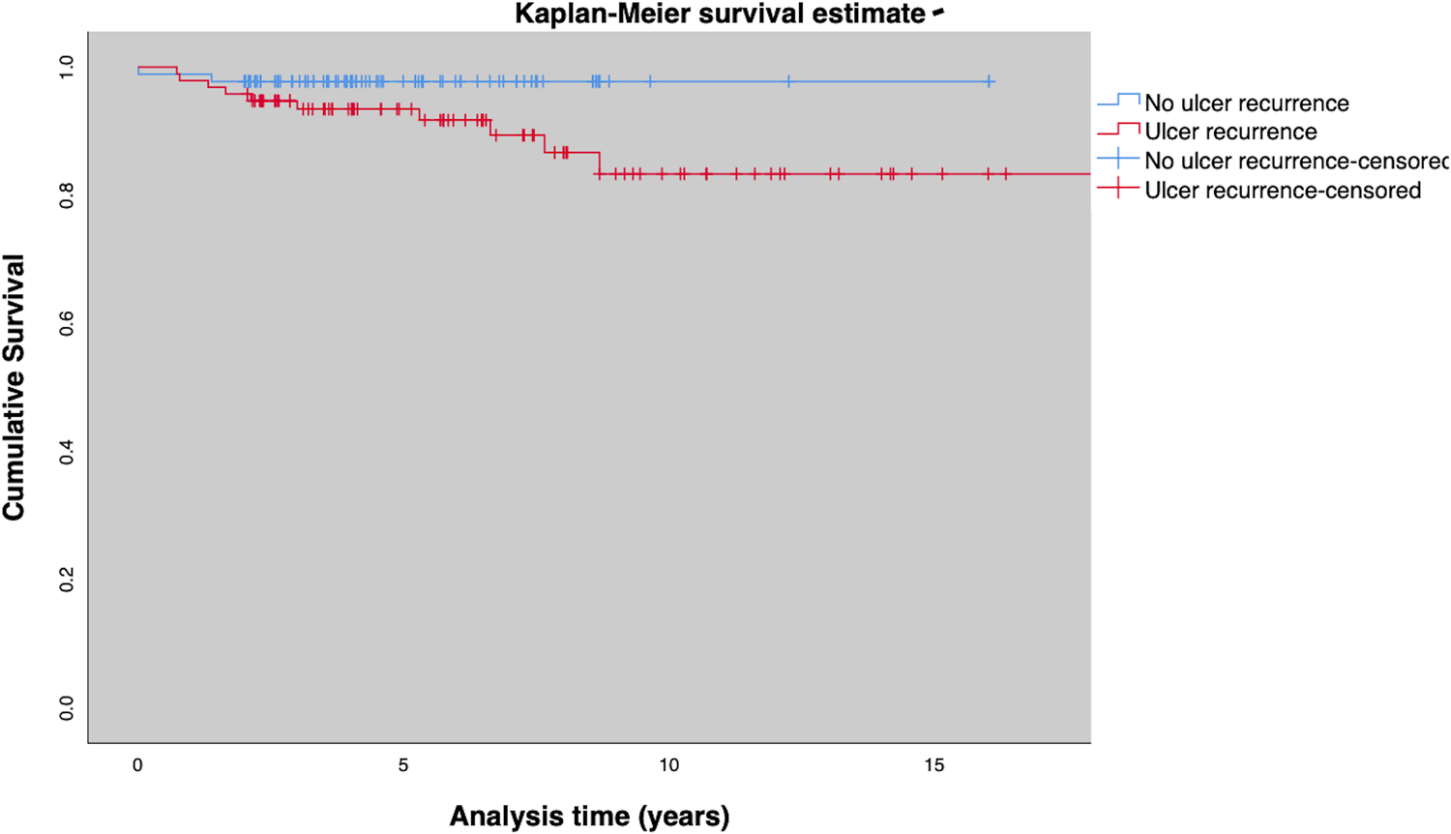
Amputation free survival for patients with and without ulcer recurrence

Surgical treatment was removal of exostoses in 10 (5.6%) feet, ulcer debridement in 30 (16.4%) feet, internal realignement arthrodesis in 8 (4.3%) feet, and one-staged external realignment arthrodesis using the Ilizarov ring fixator in 18 (9.8%) feet. In 35 (19.1%) feet, amputations had to be performed. 22 (12%) were minor amputations, 13 (7.1%) were major amputations (Fig. 3, 4, 5 and 6). Median time to major amputation was 2.1 (IQR 4.5) years. Amputation details are shown in Table 4.

**Table 4:** Summary of minor and major amputations

	Feet (*n*, %) (Cohort *n* = 184, 100%)
Minor amputations	22 (12.0%)
Toe level amputation	16 (8.7%)
Transmetatarsal amputation	4 (2.2%)
Bona-Jaeger amputation	1 (0.5%)
Pirogoff-Spitzy amputation	1 (0.5%)
Major amputations	13 (7.1%)
Transtibial amputation	13 (7.1%)
Transfemoral amputation	0 (0%)
No amputation	149 (81.0%)

### Statistical analysis

#### CN reactivation

Presence of Diabetes mellitus was significantly associated with CN reactivation (*p* = 0.022). Reactivation could be seen more frequently in type 1 diabetics (6/18; 33%) than in type 2 diabetics (15/113; 13%) or in patients suffering from CN without diabetes (5/53; 8%). All other tested variables were not statistically associated with CN reactivation (Table 5).

**Table 5:** Details of CN reactivations

	Total	CN reactivation	No CN reactivation	*P*
Feet, *n* (%)	184	25 (13.6)	159 (86.4)	
Gender				.729^a^
Women	57	7 (12.3)	50 (87.7)	
Men	127	18 (14.2)	109 (85.8)	
Age		57	60	.1548^a^
BMI		31.1	28.4	.2310^a^
Presence of diabetes				.022^a^
No diabetes	53	4 (7.5)	49 (92.5)	
Diabetes Type I	18	6 (33.3)	12 (66.7)	
Diabetes Type II	113	15 (13.3)	98 (86.7)	
Renal insufficiency	42	9 (21.4)	33 (78.6)	.091^a^
Smokers	54	6 (11.1)	48 (88.9)	.528^a^
Patients with PAD	40	7 (17.5)	33 (82.5)	.414^a^
Type of neuropathy				.803^a^
Localization, *n* (%)				.091^a^
Sanders I	25	7 (28.0)	18 (72.0)	
Sanders II	87	9 (10.3)	78 (89.7)	
Sanders III	56	9 (16.1)	47 (83.9)	
Sanders IV	11	0 (0.0)	11 (100.0)	
Sanders V	5	0 (0.0)	5 (100.0)	

^a^Pearson chi-square/Fisher’s exact test; statistical significance is indicated in bold

#### Development of contralateral CN during follow-up

Female gender (*p* = 0.014) was significantly associated with development of contral CN during follow-up (13/57 (22.8%) female feet vs 12/127 (9.4%) male feet). All other tested variables were not statistically associated with Development of contralateral CN during follow-up (Table 6).

**Table 6:** Details of contralateral CN

	Total	No contralateral CN	Contralateral CN	*P*
Feet	184	159	25	
Gender				0.014^a^
Women	57	44 (77.2)	13 (22.8)	
Men	127	115 (90.6)	12 (9.4)	
Age		61	59	0.2595^a^
BMI		28.7	28.6	0.8553^a^
Presence of diabetes				0.377^a^
No diabetes	53	48 (90.6)	5 (9.4)	
Diabetes Type I	18	14 (77.8)	4 (22.2)	
Diabetes Type II	113	97 (85.8)	16 (14.2)	
Renal insufficiency	42	35 (83.3)	7 (16.7)	0.507^a^
Smokers	54	48 (88.9)	6 (11.1)	0.528^a^
Patients with PAD	40	35 (87.5)	5 (12.5)	0.821^a^
Localization, *n* (%)				0.408^a^
Sanders I	25	24 (96.0)	1 (4.0)	
Sanders II	87	72 (82.8)	15 (17.2)	
Sanders III	56	49 (87.5)	7 (12.5)	
Sanders IV	11	9 (81.8)	2 (18.2)	
Sanders V	5	5 (100.0)	0 (0.0)	

^a^Pearson chi-square/Fisher’s exact test

#### Ulcer development

PAD (*p* = 0.049) and smoking (*p* = 0.002) were significantly associated with ulcer development. All other tested variables were not statistically associated with ulcer development (Table 7).

**Table 7:** Summary of ulcer development and ulcer recurrence

	Total	Ulcer development	No ulcer development	*P*	Total	Ulcer recurrence	No ulcer recurrence	*P*
Feet	184	55	129		184	95	89	
Gender				0.718^a^				0.438^a^
Women	57	16 (28.1)	41 (71.9)		57	27 (47.4)	30 (52.6)	
Men	127	39 (30.7)	88 (69.3)		127	68 (53.5)	59 (46.5)	
Age		59	61	0.9241^a^		57	61	0.0380^a^
BMI		28.9	28.2	0.0564^a^		28	29.1	0.7402^a^
Presence of diabetes				0.280^a^				0.023^a^
No diabetes	53	13 (24.5)	40 (75.5)		53	19 (35.8)	34 (64.2)	
Diabetes Type I	18	8 (44.4)	10 (55.6)		18	11 (61.1)	7 (38.9)	
Diabetes Type II	113	34 (30.1)	79 (69.9)		113	65 (57.5)	48 (42.5)	
Renal insufficiency	42	14 (33.3)	28 (66.7)	0.579^a^	42	24 (57.1)	18 (42.9)	0.416^a^
Smokers	54	25 (46.3)	29 (53.7)	0.002^a^	54	35 (64.8)	19 (35.2)	0.021^a^
Patients with PAD	40	17 (42.5)	23 (57.5)	0.049^a^	40	29 (72.5)	11 (27.5)	0.003^a^
Localization, n (%)				0.091^a^				0.003^a^
Sanders I	25	10 (40.0)	15 (60.0)		25	15 (60.0)	10 (40.0)	
Sanders II	87	32 (36.8)	55 (63.2)		87	54 (62.1)	33 (37.9)	
Sanders III	56	10 (17.9)	46 (82.1)		56	22 (39.3)	34 (60.7)	
Sanders IV	11	2 (18.2)	9 (81.8)		11	1 (9.1)	10 (90.9)	
Sanders V	5	1 (20.0)	4 (80.0)		5	3 (60.0)	2 (40.0)	

^a^Pearson chi-square/Fisher’s exact test

#### Ulcer recurrence

Patients developing ulcer recurrence were significantly younger (median age 57 years) than those without ulcer recurrence (median age 61 years) (*p* = 0.038). Further, smokers were more likely to develop ulcer recurrence than non-smokers (*p* = 0.021) as were patients with PAD (*p* = 0.003), and patients suffering from diabetes (*p* = 0.023). Sanders type was significantly associated with ulcer recurrence (*p* = 0.003): 56.9% feet with ulcer recurrence had Sanders type 2 deformity initially. All other tested variables were not statistically associated with ulcer recurrence (Table 7).

#### Any amputation

Diabetes (*p* < 0.001), PAD (*p* < 0.004), and smoking (*p* < 0.001) were significantly associated with the need for amputation. All other tested variables were not statistically associated with any amputation (Table 8).

**Table 8:** Summary if amputations

	Total	Any amputations	No amputations	*P*	Total	Major amputations	No major amputations	*P*
Feet	183	35	148		184	13	171	
Gender				0.113^a^				0.523^a^
Women	57	7 (12.3)	50 (87.7)		57	3 (5.3)	54 (94.7)	
Men	126	28 (22.2)	98 (77.8)		127	10 (7.9)	117 (92.1)	
Age		59	60	0.3912^a^		51	60	0.0813^a^
BMI		27.7	28.9	0.4585^a^		27.8	28.9	0.5534^a^
Presence of diabetes				0.000^a^				0.000^a^
No diabetes	53	6 (11.3)	47 (88.7)		53	1 (1.9)	52 (98.1)	
Diabetes Type I	18	10 (55.6)	8 (44.4)		18	6 (33.3)	12 (66.7)	
Diabetes Type II	112	19 (17.0)	93 (83.0)		113	6 (5.3)	107 (94.7)	
Renal insufficiency	41	11 (26.8)	30 (73.2)	0.155^a^	42	4 (9.5)	38 (90.5)	0.479^a^
Smokers	54	19 (35.2)	35 (64.8)	0.000^a^	54	8 (14.8)	46 (85.2)	0.008^a^
Patients with PAD	40	14 (35.0)	26 (65.0)	0.004^a^	40	5 (12.5)	35 (87.5)	0.129^a^
Localization, n (%)				0.688^a^				0.920^a^
Sanders I	25	6 (24.0)	19 (76.0)		25	1 (4.0)	24 (96.0)	
Sanders II	87	19 (21.8)	68 (78.2)		87	7 (8.0)	80 (92.0)	
Sanders III	55	8 (14.5)	47 (85.5)		56	4 (7.1)	52 (92.9)	
Sanders IV	11	1 (9.1)	10 (90.9)		11	1 (9.1)	10 (90.9)	
Sanders V	5	1 (20.0)	4 (80.0)		5	0 (0.0)	5 (100.0)	

^a^Pearson chi-square/Fisher’s exact test

#### Major amputation

Presence of diabetes (*p* < 0.001) and smoking (*p* = 0.008) were significantly associated with the need for major amputation. All other tested variables were not statistically associated with major amputation (Table 8).

#### Power analysis

A post hoc sample size calculation for one of the secondary outcomes, the association of major amputation and smoking yielded a power of 92.7% with an actual alpha of 2.7% (amputations in smokers = 19 (35.2%) (*n* = 54) versus amputations in non-smokers = 16 (11.5%) (*n* = 139).

## Discussion

Clinical relevance of the present study is that initial conservative treatment of Charcot Arthropathy leads to limb salvage in 92.9% of the affected feet after a median of 5.2 years follow-up.

The present study contains 159 CN patients with 184 affected feet and is not limited to CN in patients with diabetes. The patient number is the second largest in the literature to Jansen et al., who retrospectively analysed 173 patients and 176 CN feet with diabetic CN over a substantial shorter period with a mean follow-up of 2.6 years [31]. Other long-term outcome reports on CN had either fewer patients or shorter follow-up (Bariteau et al. [25], Christensen et al. [35], Fabrin et al. [4], Saltzmann et al. [30]), longer follow-up periods but substantially fewer patients than the present work (Nilsen et al. [27], Pakarinen et al. [29]) or fewer patients and inconclusive or missing information on duration of follow-up (Bergis et al. [34], O'Loughlin et al. [28]). All of the above-mentioned studies lacked one or more outcome information given in this study (Table 9). Hence, in the authors opinion, the present study contains the most comprehensive results in the literature concerning conservative treatment of CN and therefore contributes substantially to the understanding of CN`s clinical course when treated conservatively. Further, it does not preclude patients with CN developing from other reasons than diabetes. Limitations of this study are the retrospective study design with possible occurrence of a collection and/or information bias. Further, we lack a surgical control group. Also, application of total contact casts by three different nurses might have led to a treatment. Though they were trained identically, individual handling of cast techniques might have occurred. Analysis of comparable literature did not reveal information on application of total contact casts. We assume that different institutions face the same staff variability in total contact casting and therefore consider this treatment bias comparable to those of prior literature. Finally, with performance of a post hoc sample size calculation for one of the secondary outcomes, data need to be interpreted with care because this post hoc sample size calculation is not valid for all tests.

**Table 9:** Studies reporting long-term follow-up listed by mean duration of follow up

Study	Follow up (range) years	Number of patients (feet)	CA reactivation	Contralateral CA	Ulcer development	Ulcer recurrence	Any secondary surgical procedure^f^	Major amputations	Mortality rate (overall)
Nilsen [25]	8.9 (2–16)	62 (74)	n.a	17.9%	64.9%	n.a	n.a	14.9%	19.4%
Pakarinen [28]	8 (5–16)	29 (30)	n.a	2.4%	67%	40%	50%	6.7%	29.3%
Present study	6 (2–20)	159 (184)	13.6%	32.1%	72.3%	48.4%	42.4%	7.1%	13.2%
Bariteau [2]	5^a^	59 (82)^b^	n.a	39%	37%^c^	n.a	56.1%	2.4%	n.a
Fabrin [16]	4 (0.5–9.5)	115 (140)	7.1%^e^	21.7%^e^	37.9%	n.a	n.a.^g^	1.4%	1.7%
Christensen [8]	3.2^a^	56	12.5%	n.a	n.a	n.a	n.a	0%	0%^d^
Jansen [20]	2.6 (0.25–14)	173 (176)	23.3%	5.8%	67%	n.a	n.a	11.9%	39%
Saltzmann [37]	0.8 (0.5–18.5)	115 (127)	n.a	10.5%	58.5%	49%	54%	11.8%	20%

The studies of Bergis [3] and O´Loughlin [26] were excluded due to inconclusive information on duration of follow-up

Bold = highest number; n.a. = information not provided

^a^Range not provided

^b^Paper reports inconsistent numbers: it states 59 patients but lists 23 men and 37 women (= 60 patients)

^c^Only number at initial presentation given, no details over the course of follow up

^d^Death was an exclusion criterion

^e^Percentage not given but calculated based on absulte numbers provided

^f^Reflects percentage of feet, does not contain multiple procedures on the same foot

^g^6/132 (4.5%) feet had either major amputation or realignment arthrodesis, 10/108 (9.3%) patients had minor surgical procedures without information on the amount of affected feet

Reactivation of CN occurs in 12.5—29.6% [4, 31, 32, 35]. We detected reactivation clinically and radiologically (MRI) in 13.6%. The comparable study of Jansen reported of CN reactivation in 23.3% over a shorter follow-up period [31]. The difference can be explained by the fact that reactivation in Jansen´s study was defined by clinical symptoms (swelling, redness, temperature differences) alone. In our study, a positive MRI showing activity in the bones was a prerequisite for diagnosis of CN reactivation besides clinical signs. When relying on clinical symptoms alone, one may overestimate reactivation due to clinical signs of autonomic neuropathy. Most patients were detected in Eichenholtz stage 1 (*n* = 20, 80%) and most reactivations occurred at the same localization as initially, which is in accordance to previous reports [4, 32]. Median time to reactivation was 1.9 (IQR 2.2) years. Presence of Diabetes was significantly associated with CN reactivation and could be seen more frequently in type 1 diabetics (33%) than in type 2 diabetics (13%) or in patients suffering from CN without Diabetes (8%). To the best knowledge of the authors, this is new evidence and hints at different clinical courses depending on the etiology of CN. Unfortunately, Bariteau`s analysis of idiopathic CN did not contain information on reactivation which would have been interesting for comparison [25].

Reports of contralateral CN development range from 0 to 75% [4, 27, 29–32, 35–37]. The study of Clohisy with bilateral involvement of 75% reports 18 patients and thus most likely overestimates contralateral involvement [37]. In our study, 26 patients (16.4%) were affected bilaterally at the time of first CN diagnosis. During follow-up, another 25 patients (18.8%) developed contralateral CN. The median latency from diagnosis to contralateral CN was 1.1 years. At the end of follow-up, 51 patients (32.1%) were affected by bilateral CN. The slight increase compared to previous reports might be explained by longer follow-up. Further, the larger study population might have led to a more realistic result: the two studies outmatching our length of follow-up included far less patients than the present work.

Plantar pressure ulcers occur in 38–70.2% in CN [9, 27–30]. We detected ulcers in 72.3% of the affected feet at any stage of the clinical course. Most ulcers occurred at the toe or metatarsophalangeal joint level (63.2%) while the midfoot level was affected in 30%. Additionally, 6.8% had plantar heel ulcers. Presence of PAD and smoking were significantly associated with ulcer development while the Sanders type was surprisingly not. Pakarinen et al. [29] reported ulcer recurrence in 40%, while in Saltzmann et al. [30] series, 49% had ulcer recurrence and 29% even chronically recurrent ulcers. Ulcer recurrence occurred in 89 (48.4%) feet in our series. 67.4% were located at the toe level and 28.1% at the midfoot level. Younger age (perhaps reflecting more physical activity), smoking, PAD, Diabetes, and Sanders type 2 were associated with ulcer recurrence. The ulcer rate of our collective is slightly higher than in the two studies with longer follow-up (Nilsen and Pakarinen) [27, 29], which include substantially less patients and therefore might have underestimated the number of ulcers. Further, some studies reported only ulcers that derived from plantar CN-related exostoses or elevated pressure [27, 31]. Our report includes ulcers at the toe level (both plantar ulcers and dorsal mostly PIP located ulcers) as well. Substracting those, only 26.3% CN feet had at least one ulcer. Our rate of ulcer recurrence (48.4%) is within the range of previous studies. Substracting toe ulcers, the recurrence rate would be 15.8%. We attribute the low number of (recurrent) midfoot ulcers to consistent fitting of custom molded depth-insoles in 169/171 non-amputated feet.

A significant number of CN feet need any surgical procedure (30–56.1%) [25, 28–30]. In our study, 42.4% feet had at least one surgical procedure related to CN during follow-up. The median latency between first consultation and surgery was 1.5 (IQR 4.1) years. Removal of exostoses was necessary in 5.6%, and realignment arthrodesis in 14.1% (internal realignment arthrodesis: 4.3%, one-staged external realignment arthrodesis using the Ilizarov ring fixator: 9.8%). Pakarinen et al. reported of exostoses removal in 33.3% and realignment arthrodesis in 10% [29]. In Saltzman`s cohort, 54% affected feet had any surgical procedure [30]. 11 of those were exostoses removals and 14 realignment arthrodesis procedures. There was no information on possible multiple procedures on the same foot thus making percentage calculation impossible. Bariteau reported the need of surgical treatment in 56.1% of the affected feet and the absolute number of 15 realignment fusion procedures at different levels, but also failed to report if multiple realignment procedures were necessary on the same foot [25]. The slighty higher amount of realignment arthrodesis procedures might have been influenced by higher surgical experience and therefore faster indication. It could also reflect demerits in indicating conservative therapy. Both smoking and presence of a midfoot ulcer were significantly associated with the need of realignment arthrodesis. Interestingly, neither the Sanders type nor Diabetes were significantly associated with the need of realignment arthrodesis.

Overall amputation rates for patients of 20–25.7% are listed in the literature [27, 28]. In the present work, 19.1% feet needed an amputation procedure: 7.1% were major amputations and 12% minor amputations. The necessity of major amputations varies from 1.4 to 22.5% in the literature [4, 25, 27–31, 36, 38]. Fabrin’s 1.4% major amputation is not comparable to our data as it can be explained by the shorter follow-up period, and by inclusion of patients with follow-up periods of less than 2 years, who might have been amputated elsewhere [4]. Bariteau’s 2.4% might stem from the shorter follow-up and—more interestingly—from inclusion of idiopathic CN only [25]. Subanalysis of our patients with major amputation revealed that none of the 27 patients with idiopathic CN, but 9.3% of diabetic CN needed major amputation. Statistically presence of Diabetes was significantly associated with the need of major amputation besides active smoking. Neither the Sanders type nor presence of a midfoot ulcer were associated with major amputation. Thus, major amputation must be expected in any anatomical location. Elmarsafi et al. investigated risk factors for major amputation after CN reconstructive surgery [39]. They found a major amputation rate of 17.2% and identified PAD, renal disease, postoperative delayed healing (defined as nonhealing > 30 days), postoperative osteomyelitis, postreconstruction nonunions, the development of new CN sites, and increased HbA1c as risk factors for major amputation.

In comparison to surgical literature, we could find similar rates in limb salvage (92.9%) compared to studies with similar length of follow-up: 93.7% limbs could be saved according to Fragomen’s series of 16 reconstructed feet[40]. Another recent study by Spraul et al. reported a limb salvage rate of 89% while using the Hoffmann-external Fixator[41]. In a recent study with a mean of 35 months follow-up that analyzed the outcome of a one-staged reconstruction procedure using the Ilizarov principles, Wirth et al. reported successful limb salvage in 93% [42]. Ettinger et al. (31.3 months follow-up) reported a limb salvage rate of 94.8% in their series of surgically reconstructed CN ankles[43]. The same group published a case series (18 months follow-up) of tibiocalcaneal arthrodesis in CN-related talar breakdown, with 91.7% of the affected limbs being saved [44]. Pinzur et al. had a success rate of 97.3% limb salvage but had substantially less follow-up with 12 months [45]. Reinke et al. reported successful limb salvage using the Ilizarov principles in all five cases of CN with talar body necrosis after a follow-up of 27 months[46]. There were no differences in major amputations between the studies by Hegewald et al. [47], Ford et al. [48] and our study (*p* = 0.730 and *p* = 0.125, respectively; test on equality of proportions), but our study showed lower proportions of major amputations compared to Eschler et al. [49] and Elmarsafi et al. [39] (7.1% versus 28.6%, *p* = 0.030 and 7.1% versus 17.2%, *p* = 0.002, respectively). Compared to the studies by Hegewald et al. [47], Eschler et al. [49], and Elmarsafi et al. [39], our mean time to amputation was significantly different (*p* = 0.020, *p* = 0.026, and *p* = 0.006, respectively; *t* tests) with amputations occurring after a longer time frame in our study. In comparison to the study by Ford et al., our mean time to amputation showed a trend for differences (*p* = 0.056), indicating a longer major amputation-free survival as well. However, due to potential patient differences, these comparisons need to be interpreted with care.

## Conclusion

With conservative therapy of CN, limb loss can be prevented in almost 93% after 5.2 years. Patients and physicians must be prepared for a high probability of facing complications. In patients with diabetic CN, reactivation of CN is more common than in non-diabetic CN. Diabetics were at an enhanced risk of major amputation and of developing ulcer recurrence. Smoking was significantly associated with the development of ulcers and necessity of amputation procedures. Therefore, when dealing with CN, physicians must take special care if the etiology of CN is of diabetic nature and must make every effort to convince patients to stop smoking.

## Supplementary Information

Below is the link to the electronic supplementary material.

Supplementary file1 (XLSX 14 KB)

## Declarations

### Location of work statement

The study was conducted at Balgrist University Hospital, Zurich Switzerland. It served as the doctoral thesis of Ms. Gratwohl.
